# Combining multi-scale composite windows with hierarchical smoothing strategy for fingerprint orientation field computation

**DOI:** 10.1186/s12938-018-0559-4

**Published:** 2018-10-01

**Authors:** Haiyan Li, Tangyu Wang, Yiying Tang, Jun Wu, Pengfei Yu, Lei Guo, Jianhua Chen, Yufeng Zhang

**Affiliations:** 1 School of Information Science and Engineering, Electronic Engineering, Yunnan University, Chenggong District, Kunming, 650000 China; 2 Breast Surgery Department, The Third Hospital Affiliated to the Medical University of Kunming, Kunming, 605118 Yunnan Province China

**Keywords:** Fingerprint orientation field (OF), Gradient based method, Weighted multi-scale composite window, Two-digitized orientation-zone filtering, Three-digitized orientation-zone filtering

## Abstract

**Background:**

Orientation field (OF) plays a very significant role in automatic fingerprint recognition systems. Many algorithms have been proposed for the estimation of fingerprints’ OF but it is hard to solve the dilemma of correcting spurious ridge structure and avoiding singularity location deviation, especially for poor images. So far, the following drawbacks still need to be solved for OF construction methods for practical application: (1) How to adaptively choose block scales to resolve the contradiction between accuracy and anti-noise, since small scale is beneficial to accuracy but is sensitive to noise, while large scale is more resistant to noise, but the accuracy is deteriorated. (2) How to construct the genuine OF in the areas close-by singular points and to evade singularity location deviation? Current block based methods give spurious OF estimates in the area near singular points because these areas have large curvature thus the detected singular points deviate from the genuine localizations. When these singular points are used as the anchor for referencing minutiae, it makes the average error of matching or recognition even larger. Therefore, it is essentials to construct the genuine OF in the areas close-by singular points and to evade singularity deviation.

**Methods:**

To overcome the above-mentioned limitations, a novel method, combining a weighted multi-scale composite window (WMCM) with a hierarchical smoothing strategy has been proposed for the computation of fingerprint OF. This method mainly contains two procedures: the approximate OF estimation and the hierarchical OF smoothing. In the first procedure, a series of OFs are established under multiple scales of composite windows by using a gradient based method then a coarse OF is estimated using the weight of each scale determined by a squared gradient consistency. In the second procedure, the OF is first quantized into a two-digitized orientation zone and a two-orientation-zone filtering strategy is adapted to the OF blocks based on a filtering mask obtained after eliminating the isolated blocks. In the end a similar three-digitized orientation zone is performed to obtain an accurate and smooth OF. To validate the performance, the proposed method has been applied to OF computation using the FVC2004 databases and three experiments are designed. Experiment 1 aims to validate whether the weighted multi-scale composite window can balance the dilemma of accuracy and robustness more effectively than the previous works do. Experiment 2 is designed to examine whether the hierarchical smoothing method can correct the spurious ridge flow and preserve the genuine localization of singular points. The purpose of experiment 3 is to test the performance of the proposed method on OF reconstruction in low quality fingerprint images. The fingerprint databases FVC 2004 DB1–DB4 are employed in this study.

**Results:**

The results of experiment I shows that the proposed method is capable to extract the information of OF reliably and it is more robust against singularity localization deviation in comparison with the other three gradient based methods. The results of experiment II indicates that the proposed smoothing method can balance the contradiction in correcting spurious ridge structures and preserving genuine singularity localization. The results of experiment III illustrates that our approach combing WMCW with the hierarchical smoothing method is capable to extract the information of OF ridge reliably and it is more robust against singularity deviation in comparison with the other three gradient based methods. In a word, the experiment results demonstrate that the proposed method can correct spurious ridge structure and meanwhile avoid singularity deviation compared with the previous works.

**Conclusions:**

A novel gradient based algorithm has been proposed which is more reliable for the estimation of the ridge information for fingerprint OF and is more accurate in preserving the singularity localization. Compared with the previously proposed gradient based methods, the advantages of the proposed RBSF lie in three aspects. Firstly a weighted multi-scale composite window is put forward to replace the single window used by conventional gradient based methods and to adaptively choose the scales of the blocks. Secondly, a hierarchical smoothing strategy is proposed to enhance the OF by using the two-orientation-zone filtering and the three-orientation-zone filtering, aiming to correct the spurious ridges and preserving the genuine location of singular points. Finally, three experiments are designed to test the proposed algorithm together with other popular gradient based methods on real fingerprint images, which are selected from different categories and all are suffering from obvious noise effects. All the experiment results show that the proposed method is superior with respect to reliable OF construction and avoiding singularity localization deviation.

## Background

Ridge orientation pattern, representing the ridge flow directions on regularly spaced grids and revealing intrinsic features of ridge topologies, plays a critical role in singularity extraction, fingerprint classification, fingerprint recognition and so on. Therefore, a huge amount of research efforts have been made towards the reliable estimation of fingerprint orientation pattern from fingerprint images which can roughly be classified as global modeling [1–8] and local estimation [9–19].

Global fingerprint structure can be used for OF estimation in such a way that OF in bad quality areas can be accurately interpolated by using singular points as heuristic knowledge. Pioneered global modeling based method, a so-called zero-pole model, for orientation field computation based on singular points was proposed [1]. The approach modeled cores and deltas as zero and a pole in the complex plane and the orientation was computed by the summation of the influence of singularities. Since then this model receives a wide acceptance and improvements. Vizcaya and Gerhardt [2] used a piecewise linear approximation model around singular points to adjust the zero and pole’s behavior. Gu et al. [3, 4] proposed a combination model for orientation field representation, in which the global orientation is firstly constructed by a polynomial model and subsequently a point-charge model was applied to correct regions near singular points. However, these global based methods mostly depend on the accurate detection of singular points, and the precise detection of singular points, in turn, depends on the correct computation of the OF. As a result, the problem turns into the paradoxical chicken-eeg problem. In order to solve the paradox, OF estimation are treated as data fitting problems [5, 6]. Weights are assigned based on foreground [7] and background pixel or probable location of singular point [8] to evade spurious OF estimation in bad quality areas or singular points close-by areas. Even though these algorithms exhibit better performance than the methods in [5, 6], it is obvious that the success of data-fitting based global OF modeling mainly relies on accurate local OF estimates and proper weight assignment.

Comparatively, local methods do not require any prior knowledge of singular points or use neighborhoods for OF estimation. These are mainly categorized as: filter-bank based algorithm [9, 10] and gradient-based algorithm [11–19]. The filter-based methods are resistant to noise but they almost completely rely on the limited number of filters. Therefore, the results of OF computation are often not very accurate. Meanwhile, the computation cost is very high because all of the filter’s outputs need to be compared. Gradient-based methods are more accurate and subtle to characterize the OF information compared with the above mentioned methods, and thus has become one of the most popular methods for OF computation. However, the following drawbacks still need to be solved for practical application:

1. How to adaptively choose the block scales? The pioneering research into the gradient-based method was proposed in 1987 [11], where the OF was calculated by the block gradient vectors. Several improvements were made by researchers over the past years and have achieved more accurate OF, which can be found in [12–19]. However, no final conclusion has yet been reached on how to choose suitable scales for the block to resolve the contradiction between accuracy and anti-noise, since small scale is beneficial to accuracy but is sensitive to noise, while large scale is more resistant to noise, but the accuracy is deteriorated. Literature [16] proposed a composite window based method to make a balance between accuracy and anti-noise, where the composite window consists of an inner window and an outer window. Thereafter, the composite window was adopted by [17] and it is proved to be able to achieve better performance compared with the previous single window based methods. However, the composite window introduces a more complex issue on how to choose proper scales for the inner and outer windows? So far, this dilemma has not been solved and current methods rely on a huge amount of experiments to obtain experientially proper scales [16, 17]. Therefore, it is critical to find a theoretical approach on how to adaptively define the scales for composite windows.
2. How to construct a genuine OF in the areas close-by singular points and to evade singularity location deviation? In order to overcoming the defect that gradient-based methods are not robust against large scale noise, a hierarchical scheme was proposed to dynamically adjust the re-estimation resolution by using coherence, defined as the deviation between the target block’s orientation and other block’s orientation around it. If the consistency level is above a certain threshold, then the target block’s orientation is re-estimated at a lower resolution level until it is under a certain level [18]. A weighted averaging method was proposed in [19], which intended to construct redundant estimation for each target block. In addition, various improvements have been achieved to enhance the robustness against large scale noise [15–17]. However, these block-based methods give spurious OF estimates in the area near singular points because these areas have large curvature thus the singular points deviate from the genuine localization. When these singular points are used as the anchor for referencing minutiae, it makes the average error of matching or recognition even larger. Therefore, it is essentials to construct the genuine OF in the areas close-by singular points and to evade singularity deviation.

The goal of this paper is to provide an OF computation method combining a weighted multi-scale composite window with hierarchical smoothing strategy. The method basically consists of two phases: the preliminary estimation of the local region orientation by a series of weighted multi-scale composite windows, followed by a refined phase for reconstructing the ridge OF using a two-hierarchy smoothing strategy. Compared to the existing approach, our approach are some of advantages. (1) An adaptive method is proposed to determine the proper scales of the composite windows, which obviates the drawback that most of the existing algorithms depend critically on a wide range of experiments for relatively proper scales. (2) A hierarchical strategy is proposed to construct genuine OF in the areas close-by singular points and to evade singularity deviation, aiming to minimize the error when singular points are used as the reference to describe minutiae for fingerprint matching. (3) Three groups of experiments are designed to validate whether the proposed algorithm is effective in computing and refining OF. The experiment results show that the proposed method can correct spurious ridge structure and avoid singularity deviation compared with the previous works.

## Methods

**Fig. 1:**
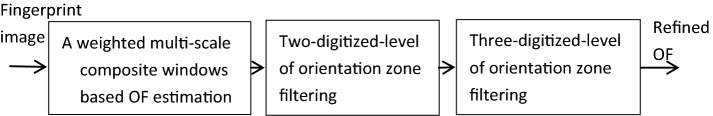
The block diagram of the proposed method

The flow-graph of the proposed fingerprint OF computation algorithm is shown in Fig. 1. It consists of two procedures. In the first procedure, a coarse OF is estimated by using a gradient based local algorithm combing a weighted multi-scale composite window. It may produce spurious ridge information for bad quality areas and singular points close-by area in the coarse OF. Subsequently, a hierarchical smoothing strategy, consisting of a two-digitized-level of orientation zone filtering and a three-digitized-level of orientation zone filtering, are performed to obtain the refined OF where OF in uniform flow area as well as the genuine singularity locations are preserved while OF in non-uniform flow areas is refined.

### Gradient based OF

In this section, we simply introduce the classical gradient-based method adopted by Kass et al. [11].

Assuming I (x, y) denotes the gray-scale values of point (x, y). The OF estimation based on the gradient method mainly contains the following steps.

1. In gradient based methods, the gradient vectors, denoted as $[G_{x} ,G_{y} ]^{T}$, are first calculated for a fingerprint image by taking the partial derivatives of gray intensity at each pixel according to Eq. (1):
  1 $$G_{x} = \frac{\partial g(x,y,\sigma )}{\partial x} \times \left( {x,y} \right) , G_{y} = \frac{\partial g(x,y,\sigma )}{\partial y} \times I\left( {x,y} \right)$$
  where $g(x,y,\sigma )$ indicates a two dimensional Gaussian core with the variance $\sigma$. $\frac{\partial }{\partial x}$ and $\frac{\partial }{\partial y}$ represent the partial derivative in *x* and *y* directions, respectively. $G_{x}$ and $G_{y}$ denote the gradient vectors in x and y coordinates, respectively. *T* represents transpose.
  A fingerprint orientation map is defined as a collection of two dimensional orientation fields. The magnitudes of these fields can be omitted. Only the angle information is of interest because it captures the dominant ridge direction in every regular spaced grid. An orientation map is commonly represented in the form of a matrix $\{ \theta_{xy} \}$, where $\theta_{xy} \in [0,\pi ]$, denoting the averaged gradient angle computed from the local gradient vectors in a grid as ${\varphi }$. In a fingerprint image, the gradient vectors always point to the directions of the highest variation of gray intensity, shown in Fig. 2, where the ridge orientation θ is orthogonal to the dominant gradient angle ${\varphi }$.

**Fig. 2:**
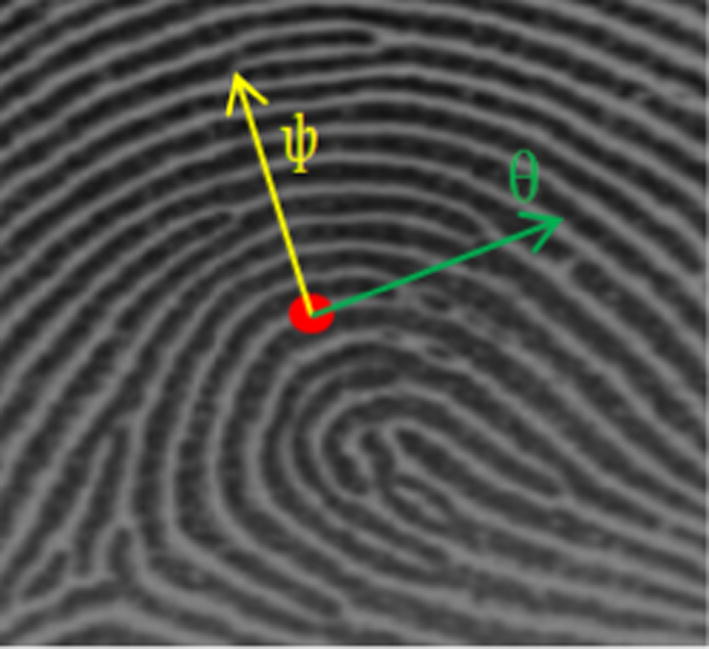
The gradient direction and the ridge direction
2. Since the ridge line has two edges, the gradient vectors at both sides of a ridge are opposite to each other. If ${\varphi }$ is calculated by averaging the gradient angles directly, the opposite gradients at both sides of the ridge line are likely to cancel each other. To solve this problem, Kass et al. [11] proposed a simple yet effective idea of doubling the gradient angles before averaging. In this way, ${\varphi }$ becomes $2{\varphi }$ and $({\varphi } + {{\uppi }})$ becomes $( 2{\varphi } + 2{\uppi})$ which is also equal to $2{\varphi }$. In practice, $2{\varphi }$ is the angle of a squared gradient vector $[G_{sx} ,G_{sy} ]^{T}$ that has the following relation with $[G_{x} ,G_{y} ]^{T}$ according to trigonometric identities:
  2 $$\left[ {\begin{array}{*{20}c} {G_{sx} } \\ {G_{sy} } \\ \end{array} } \right] = \left[ {\begin{array}{*{20}c} {G^{2} \cos 2\varphi } \\ {G^{2} \sin 2\varphi } \\ \end{array} } \right] = \left[ {\begin{array}{*{20}c} {G^{2} \left( {\cos^{2} \varphi - \sin^{2} \varphi } \right)} \\ {G^{2} 2\sin \varphi \cos \varphi } \\ \end{array} } \right] = \left[ {\begin{array}{*{20}c} {G_{x}^{2} - G_{y}^{2} } \\ {2G_{x} G_{y} } \\ \end{array} } \right]$$
3. The fingerprint image is then divided into equal-scaled and non-overlapping blocks, where *w* × *w* indicates the block scale.
4. The gradient over each block, denoted as $[G_{mx} ,G_{my} ]^{T}$, is calculated by performing average in each block independently.
  3 $$\left[ {G_{mx} ,G_{my} } \right]^{T} = \left[ {\mathop \sum \limits_{i = 1}^{w} \mathop \sum \limits_{j = 1}^{w} G_{sx} ,\mathop \sum \limits_{i = 1}^{w} \mathop \sum \limits_{j = 1}^{w} G_{sy} } \right]$$
5. Subsequently, the averaged gradient angle of each block can be calculated by using Eq. (4).
  4 $$\varphi = \frac{1}{2}\tan^{ - 1} \left( {\frac{{G_{my} }}{{G_{mx} }}} \right)$$
6. Since the ridge orientation is perpendicular to the gradient angle, therefore the ridge orientation can be obtained
  5 $$\theta = \varphi + \frac{\pi }{2}.$$

### The composite block

Since the ridge orientation is estimated in a block, there is a significant issue: how to choose a suitable scale for the block? As mentioned above, the gradient-based methods need to solve the contradiction between accuracy and robustness. If a small scale block is chosen, the orientation result is more accurate but is sensitive to noise. In contrast, if a large scale block is chosen, the robustness will be improved but the accuracy will degrade. A plain thought is choosing a suitable scale to make a balance between accuracy and robustness; however, it just makes a compromise [16].

**Fig. 3:**
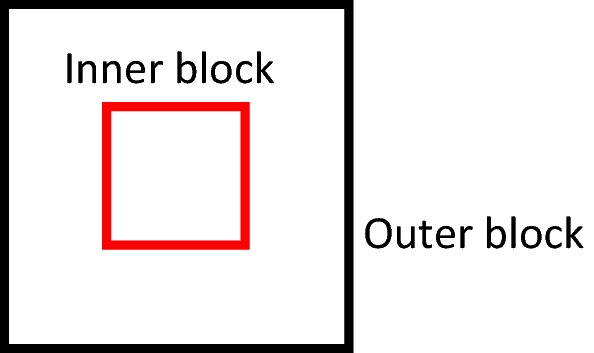
The composite window

A composite block was proposed in [16] to overcome the contradiction of accuracy and robustness. As shown in Fig. 3, the composite block consists of an inner block and an outer block, they both possess the same central point and $W_{in} \le W_{out}$. In the case that $W_{in} = W_{out}$, the composite block is equal to the single window. Therefore, the single block is just a special case of the composite block [16].

When the composite block is used for OF estimation, the orientation calculated by the outer block is set to the inner block. Since the small block is prone to accuracy and the large block is prone to robustness, the composite block combines the accuracy of a small inner block with the robustness of a large outer block. More detailed analysis can be found in [16].

**Fig. 4:**
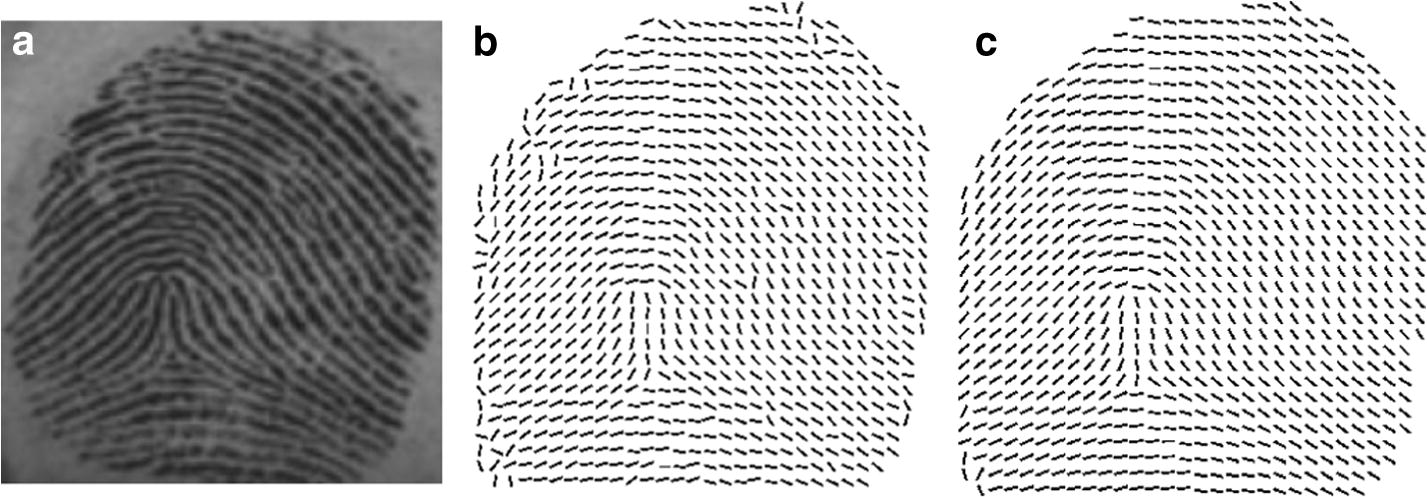
The OF estimated by using gradient based methods with a single block and a composite block: **a** the original fingerprint image, **b** the OF estimated by using a single window in 9 × 9 scale, **c** the OF estimated by using a composite window where the inner block is 9 × 9

Figure 4b presents the result calculated by using the gradient based method and the single block in 9 × 9 scale applied. Figure 4c illustrates the result calculate by using the same method and the composite block where the inner block is 9 × 9 and the outer block is 15 × 15. It is obvious that the result obtained by the composite block achieves better robustness and accuracy.

### OF estimation by using the weighted multi-scale composite block

Though the composite block integrates the robustness of a large outer block and the accuracy of a small inner block, it incurs a more complex issue: how to choose the proper scales for the inner and outer blocks? So far, this issue has not yet been theoretically solved.

**Fig. 5:**
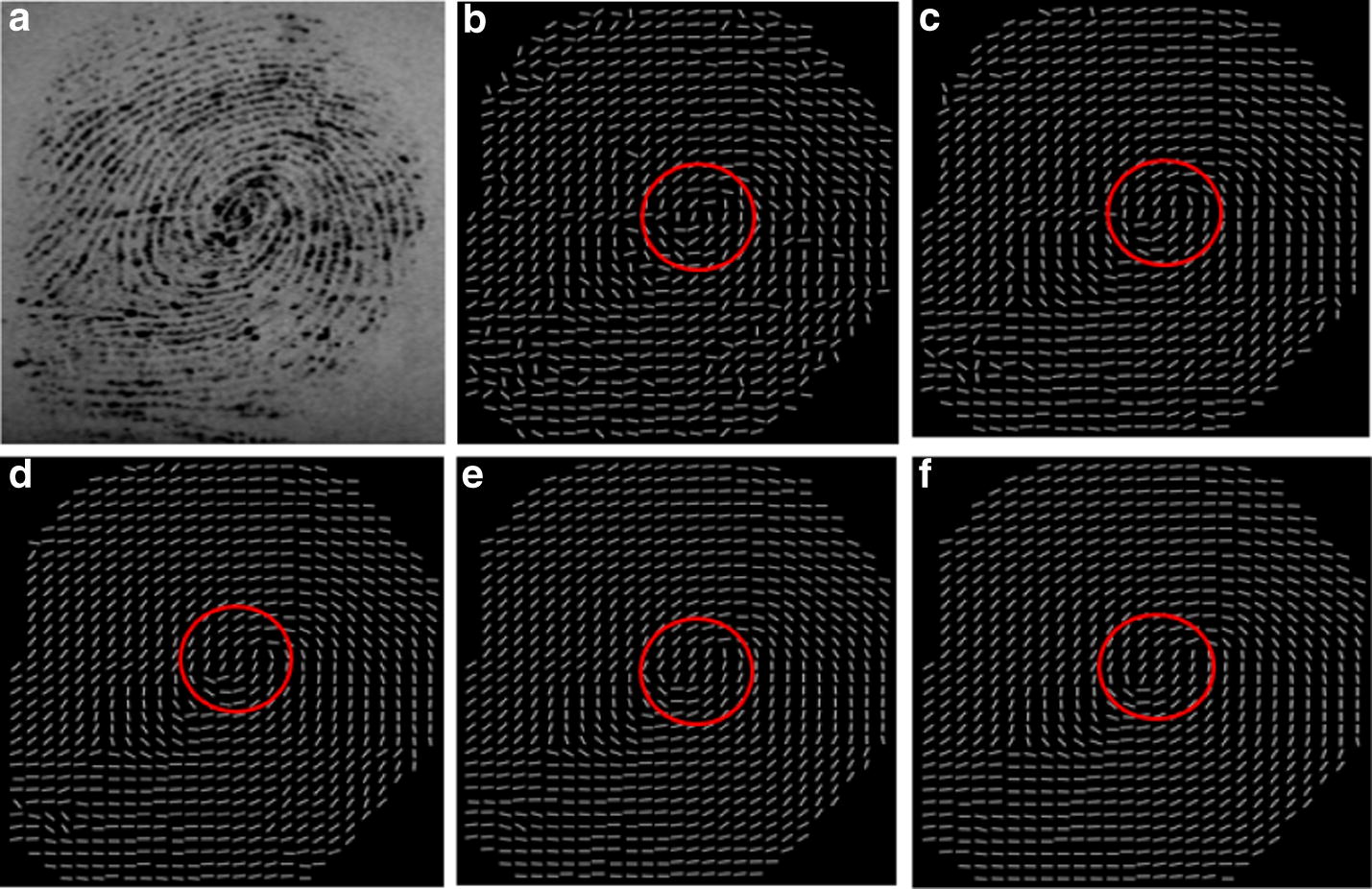
The OF estimated by using the composite block where the inner block is 9 × 9 and the outer blocks vary from small sizes to large sizes: **a** the original fingerprint image, **b** the outer block in 9 × 9 scale, **c** the outer block in 15 × 15 scale, **d** the outer block in 23 × 23 scale, **e** the outer block in 31 × 31 scale, **f** the outer block in 37 × 37 scale

In order to choose proper scales for the composite block as well as balance accuracy and robustness, OF is computed by using the composite block with the same inner block in 9 × 9 scale and the outer block in various scales. Figure 5 shows one group results of our experiment. (a) Is the original fingerprint image with poor quality and low contrast. From (b) to (f), the scales of the outer block are 9 × 9, 15 × 15, 23 × 23, 31 × 31 and 37 × 37, respectively. In (b), since the inner block and outer block have the same scales, it is essentially a single window. By observation, we can find that the result of single block, shown in (b), can preserve genuine ridge structure in singularity close by area and accurate location for singular points, marked by the red circle, while it is seriously affected by noise. With the increase of the outer block, the influence of noise comes down. However, as the scale is larger, the singularity location deviation becomes larger, shown in (d)–(f) marked in the red circles. The reason is that when the scale of outer block increases, more low frequency information is used to compute the ridge orientation, which leads to the ridge orientation seriously deviate from the genuine direction. As mentioned before, if singular points are detected with localization error, then it indicates that the estimated OF in the neighborhood of the singular point deviates from the actual OF. Consequently, the localization error of singularities will lead to incorrect results when they are used as the reference to describe minutiae for fingerprint matching.

In order to adaptively define the scales of the composite block and meanwhile balance the robustness and accuracy of preserving singularity location, a weighted multi-scale composite block is proposed. The method first computes the local OF in multiple scales of outer blocks and then a weight is assigned to the local OF in each scale based on the squared gradient coherence. The final OF is obtained by integrating OFs in multiple scales. The proposed method is described with details in the following subsection.

In order to measure the reliability of estimation, Kass et al. [11] proposed a metric called coherence. The coherence metric calculates the strength of the average gradient in the distribution of local gradient vectors. Assuming $coh(p,\;q,\;l)$ denotes the squared gradient coherence and $\theta (p,q,l)$ represents the local OF for the block where the scale of inner block is $q*q$ and the scale of outer block is $p*p$. Then the coherence is given by:

6 $$coh\left( {p,q,l} \right) = \frac{{\left| {\mathop \sum \nolimits_{i = - p/2}^{2/p} \mathop \sum \nolimits_{j = - p/2}^{p/2} \left( {G_{dx} \left( {i,j} \right),G_{dy} \left( {i,j} \right)} \right)} \right|}}{{\mathop \sum \nolimits_{i = - p/2}^{2/p} \mathop \sum \nolimits_{j = - p/2}^{p/2} \left| {\left( {G_{dx} \left( {i,j} \right),G_{dy} \left( {i,j} \right)} \right)} \right|}}$$

where $p = q + l*k$ and $l = 1,2, \ldots ,L$. *L* is the number of the scales and *k* is the difference of scales between two adjacent blocks. In the following experiments, *q* is set as 9. If the coherence value is equal to 0, it indicates the gradients are equally distributed over all directions. On the contrary, if the coherence value is equal to 1, it means all squared gradient vectors share the same orientation angle. Therefore, a large weight is assigned to the block in the scale of large coherence; in contrast, a small weight is assigned to the block in the scale of small coherence. The orientation angle of the weighted multi-scale composite block is calculated by:

7 $$\theta_{final} = \tan^{ - 1} \frac{A}{B}$$

where,

8 $${\text{A}} = \frac{coh(p,q,1)}{{\mathop \sum \nolimits_{l = 1}^{L} coh(p,q,l)}}{ \sin }\left( {2\uptheta({\text{p}},{\text{q}},1)} \right) + \frac{coh(p,q,2)}{{\mathop \sum \nolimits_{l = 1}^{L} coh(p,q,l)}}{ \sin }\left( {2\uptheta({\text{p}},{\text{q}},2)} \right) + \cdots + \frac{coh(p,q,L)}{{\mathop \sum \nolimits_{l = 1}^{L} coh(p,q,l)}}{ \sin }\left( {2\uptheta({\text{p}},{\text{q}},{\text{L}})} \right)$$

9 $${\text{B}} = \frac{coh(p,q,1)}{{\mathop \sum \nolimits_{l = 1}^{L} coh(p,q,l)}}\cos \left( {2\uptheta\left( {p,q,1} \right)} \right) + \frac{coh(p,q,2)}{{\mathop \sum \nolimits_{l = 1}^{L} coh(p,q,l)}}\cos \left( {2\uptheta\left( {p,q,2} \right)} \right) + \cdots + \frac{coh(p,q,L)}{{\mathop \sum \nolimits_{l = 1}^{L} coh(p,q,l)}}\cos \left( {2\uptheta\left( {p,q,L} \right)} \right)$$

### The hierarchical OF smoothing

In general, the ridge flows are slowly varied across a fingerprint image except at several singular points while the ridge flows vary abruptly in the area close-by the singularities. Due to the difference of singularity close-by area and non-singularity area, in some extent, the method based on composite block can solve the contradiction compared with single block. The composite block performs well in the local region of small noise, but its performance deteriorates in the local region of some big fracture or holes. The reason is that the outer block is not large enough to cover the entire noise area. To compensate this issue, we can increase the scale of the outer block, however as mentioned above, the outer block in large scale leads to localization error of singular points. Another solution is to increase the number of the scales *L* or the difference of scales *k*, defined in Eqs. (6)–(9) while the computation cost is also magnified. Therefore, in order to smooth the estimated OF to obtain a refined OF, a hierarchical smoothing method is proposed which can correct the spurious ridge flow and preserve the genuine localization of singular points. The details of the proposed smoothing method are introduced as follows.

**Fig. 6:**
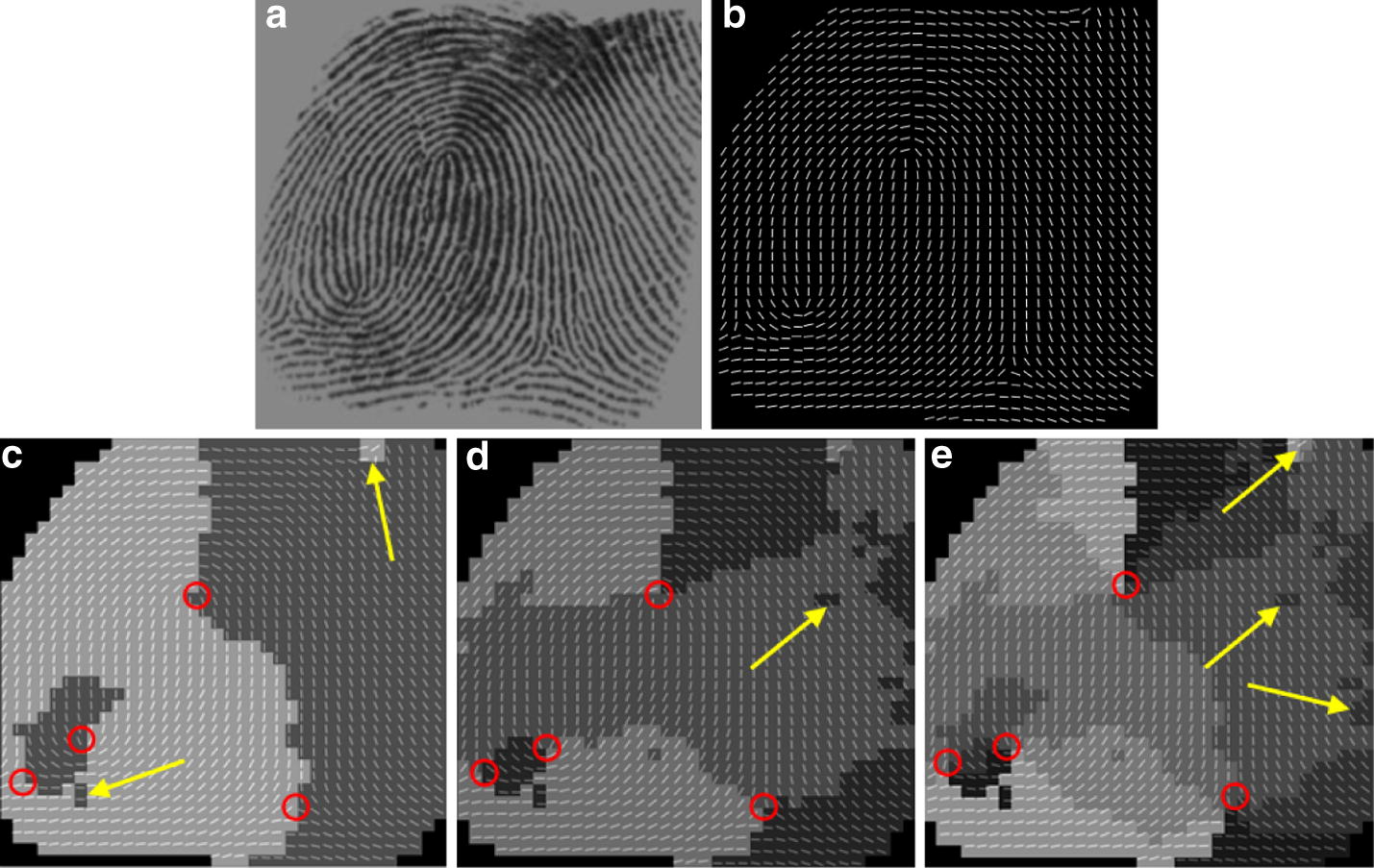
Orientation zones: **a** the original fingerprint image, **b** the OF of (**a**), **c** the orientation zone digitized as two gray scales, **d** the orientation zone digitized as three gray scales, **e** the orientation zone digitized as six gray scales

In order to visualize the directional image OF, the directions are colored in gray scales, black for 0 and white for *N* − 1. The rest of the directions *n* < *N* − 1 are represented by various gray scales. The resulting pseudo OF consists of a set of uniformly-colored regions, each called an orientation zone. Figure 6 illustrates how the number of directions applied can significantly affect the information of the orientation zones. Figure 6a is the original fingerprint image. (b) is the OF corresponding to (a). (c–e) Are the illustrated orientation zones digitized as two, three and six gray scales, respectively. A border line is defined as the separating boundary between two adjacent orientation zones. In other words, a border line is where the ridges’ digitized orientation changes.

Several properties exist when an OF is quantified into two or more orientation zones.

#### **Property 1**

*Singular points only locate at the intersection of border lines.*

#### *Proof*

The intersections of border lines are marked by red circles in Fig. 6c–e. Let Ω_*i*_ and Ω_*j*_ be two distinct border lines, which Ω_*i*_ is the boundary between Ω(n) and Ω(n + 1) as well as Ω_*j*_ is the boundary between Ω(n + 2) and Ω(n + 3), where Ω(n) is defined as the orientation zone of direction *n*. Assume that Ω_*i*_ intersects Ω_*j*_ at point X and X is not a singular point, since Ω_*i*_ and Ω_*j*_ are different border lines and intersect each other, there should be more than two distinct directions among $\{ n,\;n + 1,\;n + 2,\;n + 3\}$. If there are only two directions in the set, then Ω_*i*_ and Ω_*j*_ must be parallel. In addition, this set cannot have four distinct directions because it is assumed that Ω_*i*_ and Ω_*j*_ intersect. Therefore, there must be one region in common among Ω(n), Ω(n + 1), Ω(n + 2) and Ω(n + 3), indicating that X is the center of a cycling domain. Therefore, X is a singular point according to the definition of singular points, which a core point is defined as a concentrate region where the region curvature is converging to a local maximum and a delta point is defined as a region where the ridge curvature is converging to a local minimum [20].

#### **Property 2**

*An island of discontinuities, called a hole, is noise if it is not on the border lines or close to the border lines.*

#### *Proof*

Holes are marked by yellow arrows in Fig. 6c–e. According to the characteristics of noise and edge, edges and noise may be defined as sharp changes in intensity in gray level images [21]. That is, the intensity of noise and edge pixel varies sharply from the neighboring pixels’ intensity. Therefore, an island of discontinuities is defined as noise if it is not on the border lines or close to the border lines because a border line is defined as the separating boundary between two adjacent gray scales.

#### **Property 3**

*When the number of gray levels, denoted as N, increases, the singular points become out of focus.*

#### *Proof*

According to the definition of orientation zone, the number of gray level *N* is equivalent to the number of orientation. Compared Fig. 6e with (c) and (b), it is observed that the number of *N* greatly affect the number of border line presented. If = 1, there will be no pattern at all. If *N* = 2, then ridges will be bisected and no intersection can be formed. Therefore, we conclude that the orientation number *N* should be at least 3. However, when $N \ge 4$, the number of border lines becomes more and the singular points become out of focus with the increase of *N*.

Based on the three properties, we propose a hierarchical smoothing method, which the OF is digitized into two-orientation-zone in the first step and the OF is digitized into three-orientation-zone in the second step. The propose method does not digitize the OF into four or more direction zones since the singular points become out of focus with the increase of *N*. The details of the proposed method are introduced as follows.

The Gaussian low pass filter is applied for smoothing the fingerprint image, which is defined as:

10 $$g\left( {x,y} \right) = \exp \left( {\frac{{x^{2} + y^{2} }}{{2\delta^{2} }}} \right).$$

When processing digital fingerprint images, Eq. (10) is digitized as:

11 $$g\left( {x,y} \right) = \exp \left( {\frac{{\left( {x - i} \right)^{2} + (y - j)^{2} }}{{2\delta^{2} }}} \right).$$

where (*i*, *j*) is the center of the Gaussian kernel. Since 90% percent of the energy is located in $( - 2\updelta,\;2\updelta)$ in terms of the Gaussian low pass filter with the variance of $\updelta$. Therefore, $( - 2\updelta,\;2\updelta)$ is set as the dominant area of the Gaussian low pass filter. For example, when $\updelta = 1.0$, the Gaussian kernel can be computed as:

12 $$\left[ {\begin{array}{ccccc} {0.0183} & {0.0821} & {0.1353} & {0.0821} & {0.0183} \\{0.0821} & {0.3679} & {0.6065} & {0.3679} & {0.0821} \\ {0.1353} &{0.6065} &{1}& {0.6065} & {0.1353} \\{0.0821} &{0.3679} &{0.6065} &{0.3679} &{0.0821} \\{0.0183} & {0.0821} &{0.1353} &{0.0821} &{0.0183} \end{array} }\right]$$

### Two-orientation-zone filtering

The OF is first digitized as two equivalent directional zones where $\omega_{1}^{1}$ and $\omega_{2}^{1}$ represent the direction zones whose angle is (0, 90°) and [90°, 180°], respectively. Subsequently, holes are removed as noise if they are not on the border lines. The pixel number of the two-orientation-zone, denoted as $\omega_{1}^{1}$ and $\omega_{2}^{1}$ in the filtering window $S_{n}^{1} \times S_{n}^{1}$ are counted and represented as $num_{n1}^{1}$ and $num_{n2}^{1}$, respectively. Assuming $Num_{n}^{1}$ is the maximal value of $num_{n1}^{1}$ and $num_{n2}^{1}$, then the variance of the Gaussian low pass filter for the two-orientation-zone filter, denoted as $\delta^{1}$ can be defined as:

13 $$\delta^{1} = \left\{ \begin{array}{lll} 1.5 &\quad if &\;\;Num_{1}^{1} \ge threshold_{1}^{1} \\ 1.0&\quad else\;if&\;\;Num_{2}^{1} \ge threshold_{2}^{1} \\ 0.5&\quad else\;if&\;\;Num_{3}^{1} \ge threshold_{3}^{1} \\ empty&\quad else & \\ \end{array} \right.$$

where $\delta^{1} = empty$ represents no filtering is performed. For the two-orientation-zone filter, the filter window $S_{n}^{1} \times S_{n}^{1}$ is set as $S_{1}^{1} = 7$, $S_{2}^{1} = 5$ and $S_{3}^{1} = 3$ where $n = 1,2,3$, $threshold_{n}^{1} = \text{int} (S_{n}^{1} \times S_{n}^{1} \times 0.8)$ and $\text{int} ( \bullet )$ indicates the rounding.

### Three-orientation-zone filtering

The OF is first digitized as three equivalent directional zones where $\omega_{1}^{2}$, $\omega_{2}^{2}$ and $\omega_{3}^{2}$ represent the orientation zones whose angle is within (0, 60°), [60°, 120°) and [120°, 180°], respectively. Subsequently, holes are removed as noise if they are not on the border lines. The pixel number of the three-direction-zone, denoted as $\omega_{1}^{2}$, $\omega_{2}^{2}$ and $\omega_{3}^{2}$ in the filtering window $S_{n}^{2} \times S_{n}^{2}$ are counted and represented as $num_{n1}^{2}$, $num_{n2}^{2}$ and $num_{n3}^{2}$, respectively. Thereafter, the sum of every two-orientation-zone are calculated, that is, $sum_{n1}^{2} = num_{n1}^{2} + num_{n2}^{2}$, $sum_{n2}^{2} = num_{n1}^{2} + num_{n3}^{2}$ and $sum_{n3}^{2} = num_{n2}^{2} + num_{n3}^{2}$. Assuming $Num_{n}^{2}$ is the maximal value of $sum_{n1}^{2}$, $sum_{n2}^{2}$ and $sum_{n3}^{2}$, then the variance of the Gaussian low pass filter for the three-orientation-zone filter, denoted as $\delta^{2}$ can be defined as:

14 $$\delta^{2} = \left\{\begin{array}{lll} 1.0&\quad if&\;\;Num_{1}^{2} \ge threshold_{1}^{2} \\ 0.5&\quad else\; if &\;\;Num_{2}^{2} \ge threshold_{2}^{2} \\ empty&\quad else & \end{array} \right.$$

For the three-orientation-zone filter, the filter window $S_{n}^{2} \times S_{n}^{2}$ is set as $S_{1}^{2} = 5$ and $S_{2}^{2} = 3$ where $n = 1,2$ and $threshold_{n}^{2} = \text{int} (S_{n}^{2} \times S_{n}^{2} \times 0.9)$.

## Experimental results and discussions

To evaluate the performance of the proposed weighted multi-scale composite block and the hierarchical smoothing method for OF reconstruction, three experiments are designed. Experiment 1 aims to validate whether the weighted multi-scale composite block can balance the dilemma of accuracy and robustness more effectively than the previous work. Experiment 2 is designed to examine whether the hierarchical smoothing method can correct the spurious ridge flow and preserve the genuine localization of singular points? The purpose of experiment 3 is to test the performance of the proposed method combining the weighted multi-scale composite blocks with the hierarchical smoothing strategy for OF reconstruction in low quality fingerprint images. The fingerprint databases FVC 2004 DB1–DB4 are employed in this study. All the test images suffer from the different large noise effects caused by wounds, scars, dirtiness, creases, moisture or greasiness. The experiments are carried out in Visual Studio2010+OpenCV2.4.4 and Windows 7. Due to the limited space, a part of the experimental results are presented in this session for discussion.

### OF reconstruction by using the weighted multi-scale composite block

**Fig. 7:**
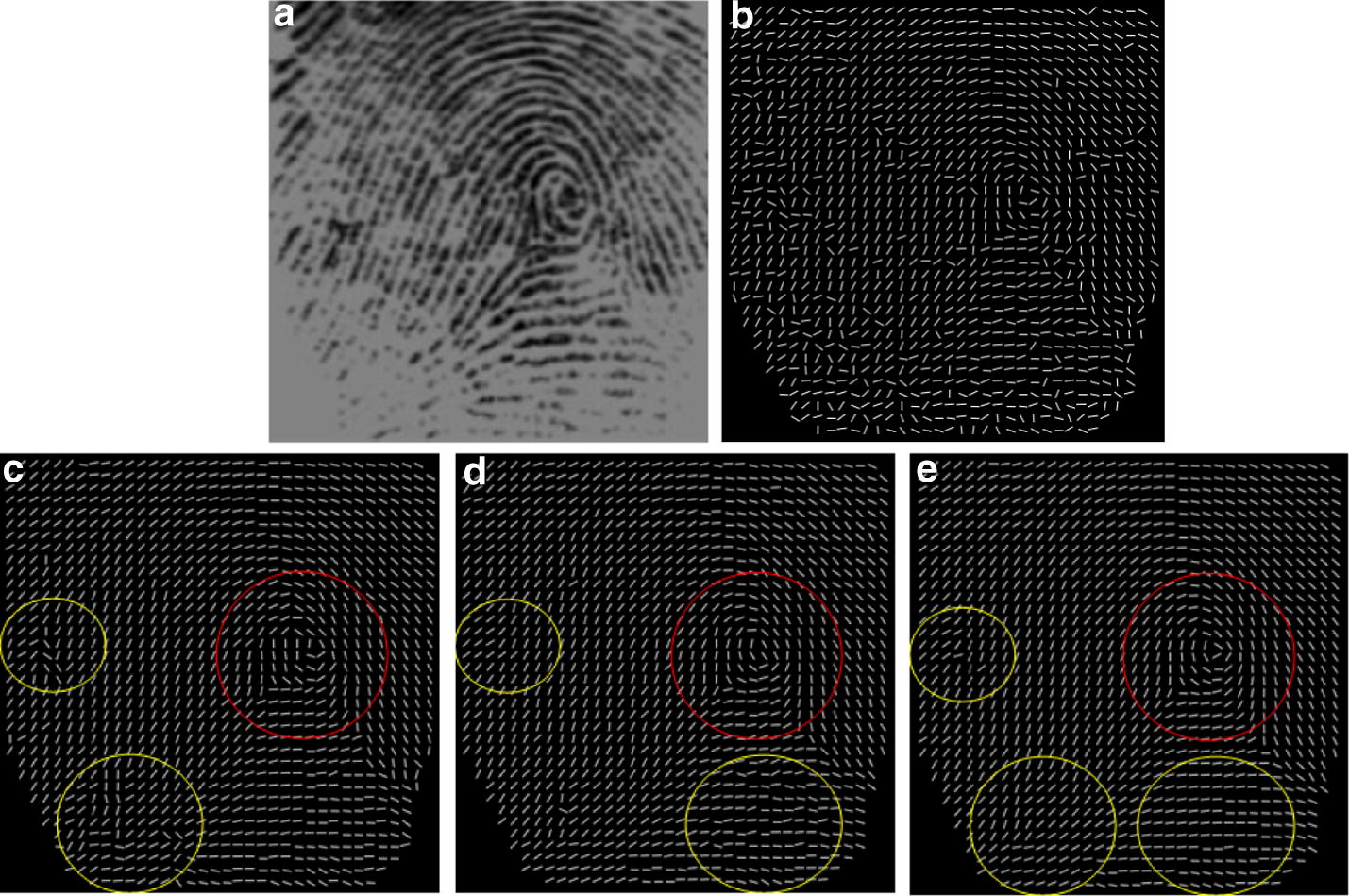
The fingerprint OF reconstruction: **a** original image affected by dryness, **b** result of the conventional gradient based method [13], **c** result of the enhanced gradient based method [23], **d** result of the gradient based and voting method [22] and **e** result using the proposed algorithm

**Fig. 8:**
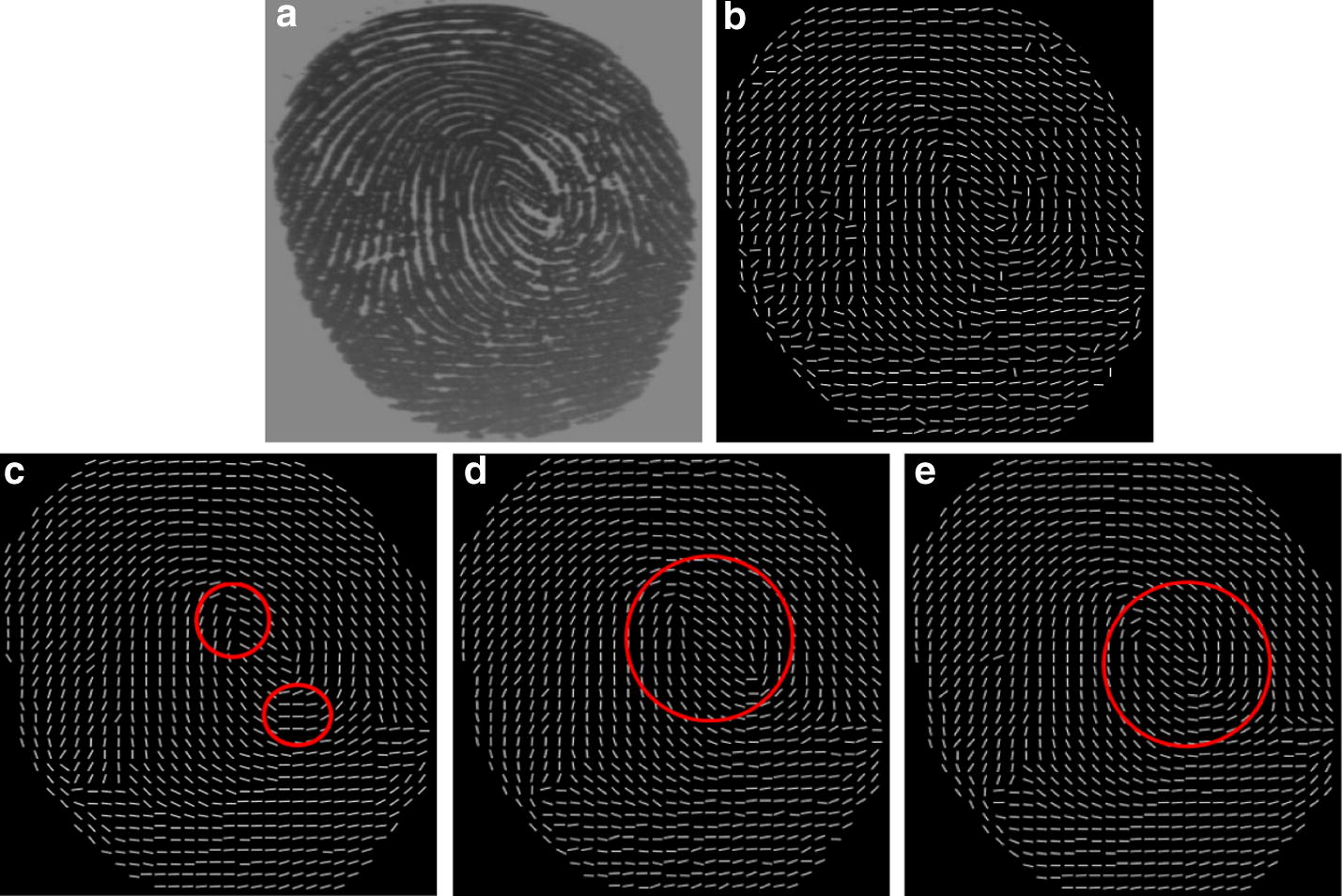
The fingerprint OF reconstruction: **a** original image affected by moisture, **b** result of the conventional gradient based method [13], **c** result of the enhanced gradient based method [23], **d** result of the gradient based voting method [22] and **e** result using the proposed algorithm

In order to examine the performance of the weighted multi-scale composite block, the proposed approach is compared with three state-of-the-art methods according to their performance in gradient based OF computation. The fingerprint OF are reconstructed by the conventional gradient based method (CG) [13], the enhanced gradient based method (EG) [23], the gradient based voting method (GV) [22] and the proposed method. The results of the four methods are shown in Figs. 7, 8. The images scale of the original fingerprints are resized as 440 × 440 for all the experiments. For the methods using single block, the scale of the block is set as 9 × 9. For the proposed method using composite block, the scale of inner is set as 9 × 9 and the scale of the outer block is defined by Eqs. (7)–(9).

Figure 7 shows the experiment results of several OF computation methods. (a) Is the original fingerprint image affected by serious level of dryness. There are lots of breakpoints caused by dryness in the ridges and the ridge information in most regions of the original image is unclear, such as the left, bottom and right regions. (b) Shows the result obtained by using the conventional gradient based method [13], obviously, the result is seriously sensitive to noise. There is no reliable OF information in (b) and the ridge flows are completely incorrect in the left, right and bottom regions since these regions are too noisy. (c) Illustrates the result estimated by using the enhanced gradient based method [23]. It is observed that the reconstructed OF is more accurate than (b) in small scale noise, but for the low quality area containing large scale noise, it is very difficult to obtain the accurate OF, marked by the yellow circle. Furthermore, it produces spurious ridge in the singular point area, marked by the red circle. Although the estimation is improved by using the gradient based voting method, shown in (d), the OF contains incorrect ridge flow in the lower left part, marked by the yellow circle, and it is noted that the singularity localization is inevitably deviated from the genuine place, marked by the red circle. Among the four, the proposed method performs the best by recovering the ridge structures, marked by the yellow circle, and preserving genuine singularity localization, marked by the red circle, shown in (e).

Figure 8a is the original fingerprint image which presents a number of indistinguishable ridge structures in the lower part due to moisture. The conventional gradient based method can only give a coarse OF containing lots of spurious ridge structures, shown in (b). (c) Is the estimation result from the enhanced gradient based method, where incorrect ridge structures are generated in the area close by the singular points, marked by the circles. While the result of the gradient based voting method incurs serious deviation for the singularity localization, shown in (d). For this fingerprint example, the result shows that the proposed method is capable to extract the information of OF reliably and it is more robust against singularity localization deviation in comparison with the other three gradient based methods.

### OF smoothing by using the two-hierarchical smoothing method

The proposed smoothing approach aims to reconstructing correct ridge structures and preserving genuine singularity localizations. We first introduce the proposed hierarchical smoothing approach in details.

**Fig. 9:**
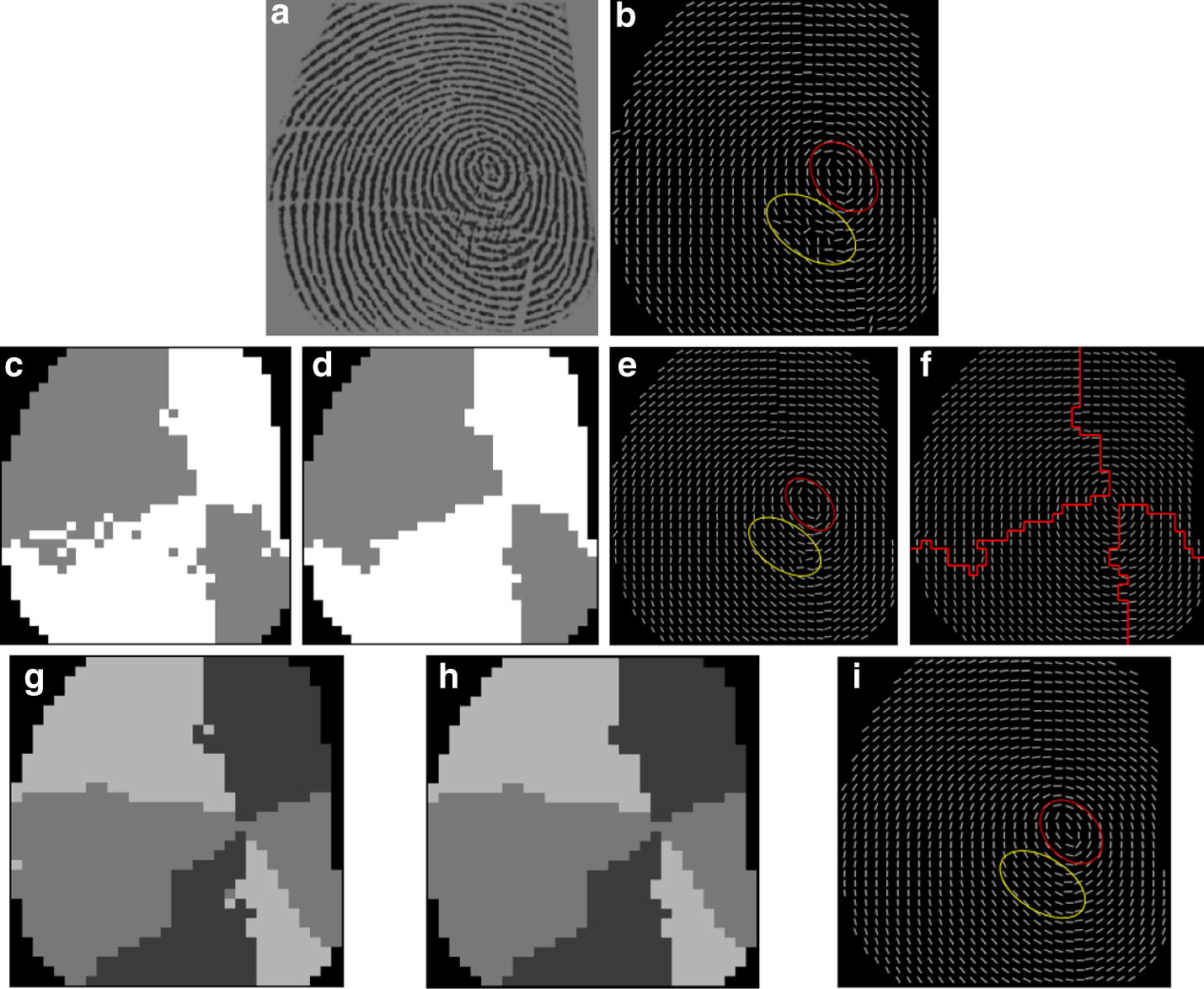
The fingerprint OF smoothing: **a** original image affected by several horizontal creases, **b** result of the conventional gradient based method [13], **c** the OF digitized as two-orientation-zones, **d** the filtering result of (**c**) after removing holes, **e** the result of two-orientation-zone filtering, **f** the border lines of the two-orientation-zone, **g** the OF digitized as three-orientation-zone, **h** the filtering result of (**g**) after removing holes, **i** the result of three-orientation-zone filtering

Figure 9b illustrates the OF estimated by using the conventional gradient based method, where there are several horizontal creases of different lengths running through the original fingerprint image, shown in (a). It is observed that spurious ridge structures are generated in the area close to the singular points due to noise, marked by the yellow circle. The purpose of our hierarchical smoothing method is to correct the spurious structures and to preserve the genuine singularity localization un-deviated, marked in the red circle. (c) Shows the OF digitized as two-orientation-zone and (d) is the corresponding result of (c) after removing holes. The result of two-orientation-zone filtering is demonstrated in (e). Comparing (b) and (e) from the figure, it can be observed that there are two significant improvements. One is that the entire OF smoothed by the two-orientation-zone filter is more consistent with respect to the actual flow of the fingerprint ridges. The other is that the spurious ridge structure is partially corrected in the area close to the singular points, marked in the yellow circle and the singular area is not affected, marked in the red circle.

According to the aforementioned Properties 1 and 2, it can be inferred that singular points must be localized along the border line when OF is digitized as two-orientation-zone. Therefore, in order to preserve the singular area unaffected, the two-orientation-zone filtering is not performed along the border lines, shown in (f). To reconstruct the actual ridge flow along the border lines, the three-orientation-zone filtering is subsequently applied.

Figure 9g shows the OF digitized as three directional zones and (h) is the corresponding result of (g) after removing holes. The result of the three-orientation-zone filtering is demonstrated in (i). Compared with the result of two-orientation-zone filtering, the spurious ridge flow close by the singular points is completely corrected, marked in the yellow circle and the singular area is nearly perfect.

**Fig. 10:**
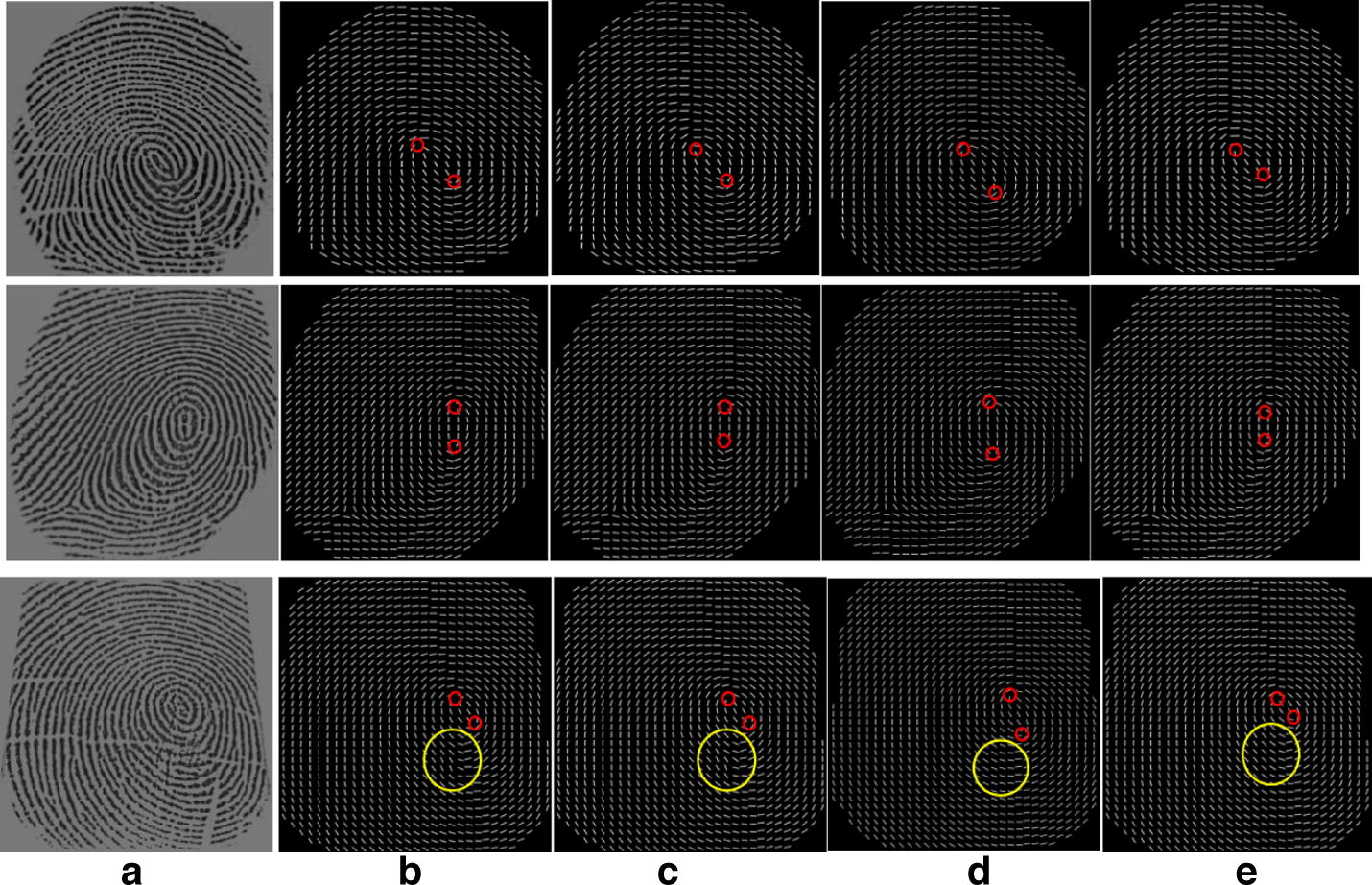
Comparison of three smoothing algorithms: **a** the original fingerprint images, the results by using **b** the variational formulation [24], **c** the adaptive smoothing method [25], **d** orientation diffusion method [26], **e** the proposed method

To test the performance of the hierarchical smoothing algorithm, the proposed method is compare with three state-of-the-art methods according to their performance in OF enhancement. The fingerprint OF are smoothed by the variational formulation by Hou [24], the adaptive smoothing method proposed by Liu [25], the orientation diffusion method proposed by Bian [26] and the proposed method. The results of the methods are shown in Fig. 10.

Figure 10a shows three examples of fingerprint images containing several vertical and horizontal scars of various lengths. The localizations of the singular points are highlighted by red circles in the estimated OFs. For these fingerprint examples, the results illustrate that the OFs estimated by using the variational formulation, shown in (b), and the adaptive smoothing method, shown in (c) produce distinct singularity localization deviation, while the proposed method can preserve genuine singularity localization. Furthermore, our proposed method is superior to the variational formulation method and the adaptive smoothing method in terms of correcting the perturbation, shown in the area marked by the yellow circles in the third line. The orientation diffusion method [26] can greatly improve the distortion shown in the area marked by the yellow circles in the third line. Unfortunately, the detected singularities obviously deviate away the genuine location due to the break line close to the singularity. Therefore, it is concluded that our proposed smoothing method can balance the contradiction in correcting spurious ridge structures and preserving genuine singularity localization.

### OF reconstruction by combining the weighted multi-scale composite blocks with the hierarchical smoothing strategy

**Fig. 11:**
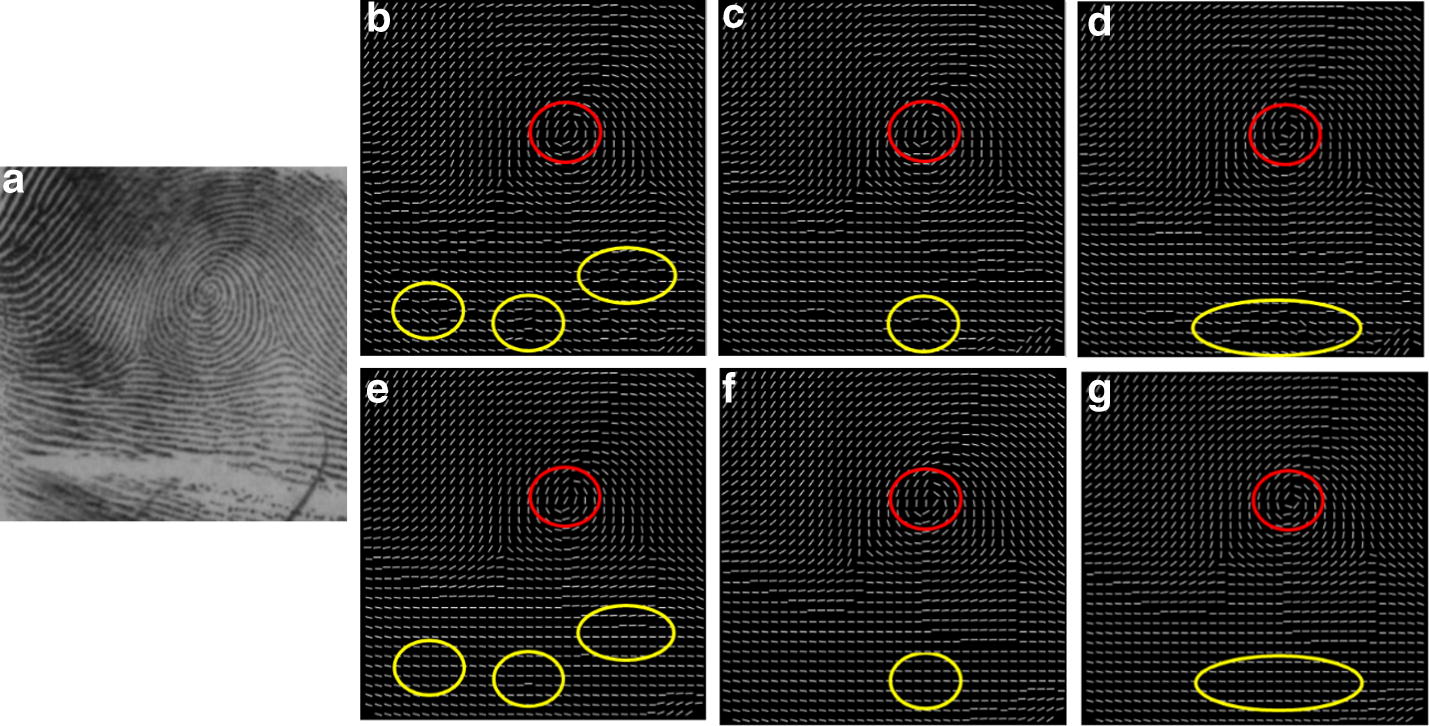
The comparison of OF construction and smoothing I: **a** original fingerprint image, OF estimated **b** by the enhanced gradient based method [23], **c** by the gradient based voting method [22], **d** by the proposed weighted multi-scale composite window (WMCW), **e** by EG + hierarchical smoothing, **f** by GV + hierarchical smoothing, **g** by the proposed WMCW + hierarchical smoothing

**Fig. 12:**
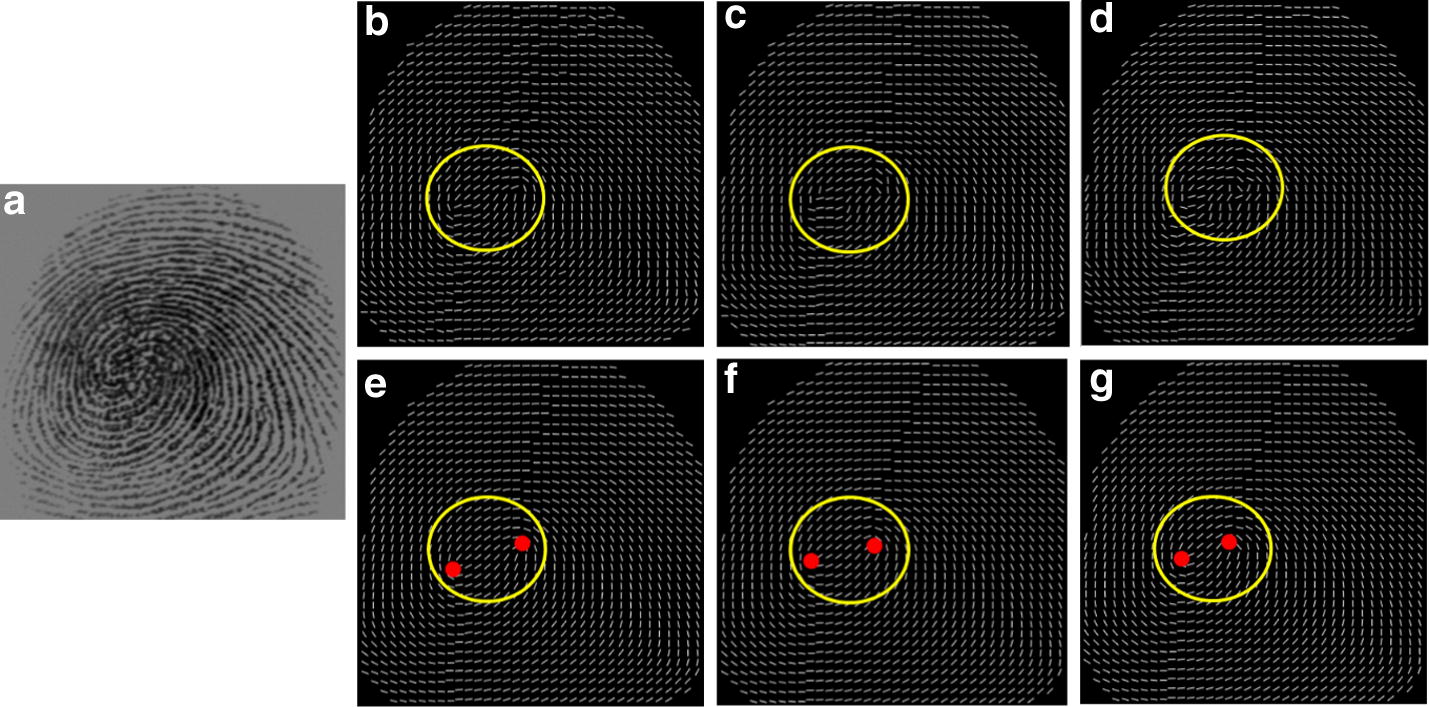
The comparison of OF construction and smoothing II: **a** original fingerprint image, OF estimated **b** by the enhanced gradient based method [23], **c** by the gradient based voting method [22], **d** by the proposed weighted multi-scale composite window (WMCW), **e** by EG + hierarchical smoothing, **f** by GV + hierarchical smoothing, **g** by the proposed WMCW + hierarchical smoothing

**Fig. 13:**
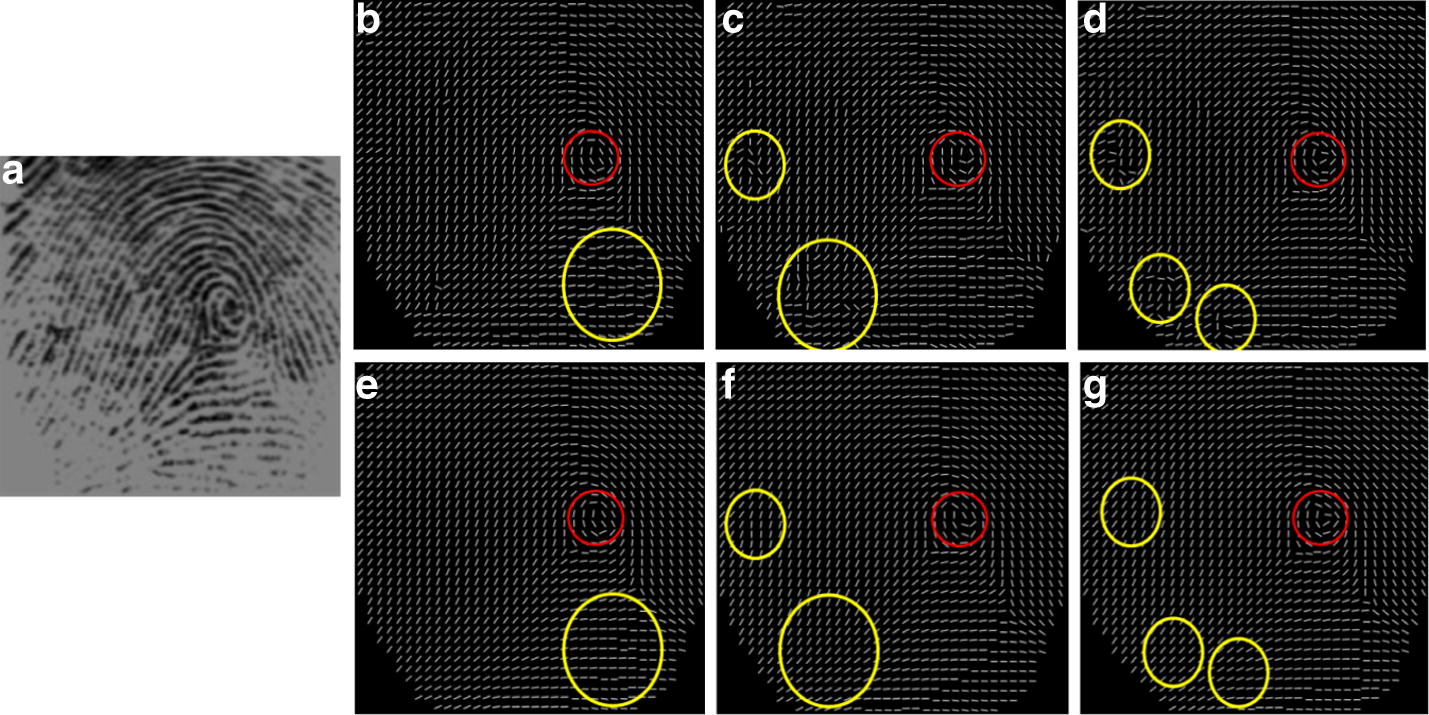
The comparison of OF construction and smoothing III: **a** original fingerprint image, OF estimated **b** by the enhanced gradient based method [23], **c** by the gradient based voting method [22], **d** by the proposed weighted multi-scale composite window (WMCW), **e** by EG + hierarchical smoothing, **f** by GV + hierarchical smoothing, **g** by the proposed WMCW + hierarchical smoothing

**Fig. 14:**
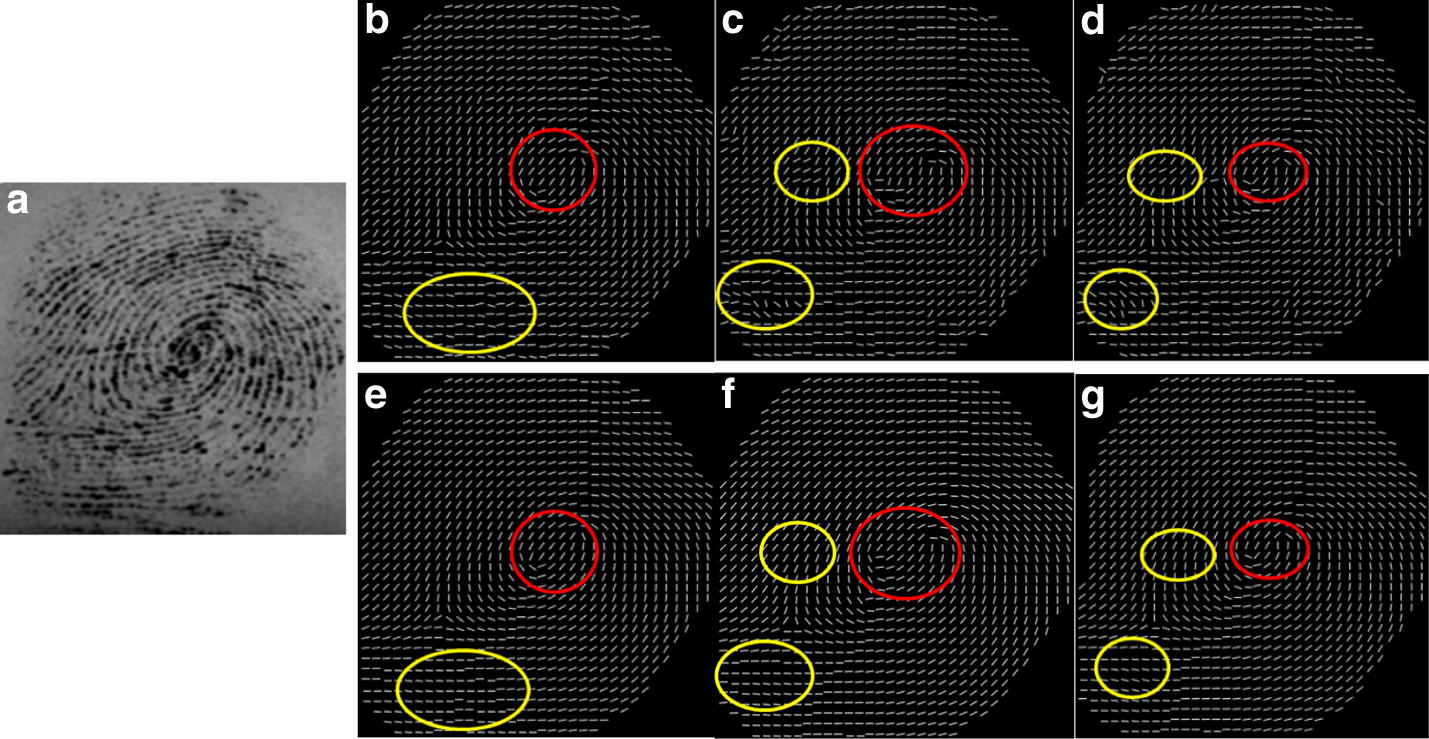
The comparison of OF construction and smoothing IV: **a** original fingerprint image, OF estimated **b** by the enhanced gradient based method [23], **c** by the gradient based voting method [22], **d** by the proposed weighted multi-scale composite window (WMCW), **e** by EG + hierarchical smoothing, **f** by GV + hierarchical smoothing, **g** by the proposed WMCW + hierarchical smoothing

Different OF estimation methods based on gradient are applied to the sample fingerprint images of low quality that are selected from the database FVC2004. These fingerprint samples are affected by serious level of dirtiness, creases, moisture or dryness. Figures 11, 12, 13 and 14 show that the performance comparison for the OF reconstruction by using different OF extraction methods. For the purpose of comparison, the estimated spurious ridge structures are marked in yellow circles and the area of singular points are marked in red circles.

From Fig. 11a we can observe that a long and wide blotch, possibly caused by callus, appears at the lower site of the original image and the ridge pattern in this area is completely lost. We then superimpose the estimated OF from different gradient based methods on the original fingerprint image and display the results in Fig. 11b–d. (b) and (c) Are produced from the enhanced gradient based method (EG) and the gradient-based voting method (GV), where several spurious ridge structures are contained. Although the estimation of the proposed weighted multi-scale composite window (WMCW) generates incorrect ridge flow, the OF are the smoothest among the results of the three methods. From (e) to (g) we see the spurious ridge structures are correctly reconstructed by using the proposed hierarchical smoothing method. The red circles in the figure indicate the singular areas. One can see that the proposed WMCW combining the hierarchical smoothing method performs the best by preserving genuine singularity localization, shown in (d) and (g), while the other methods produce singularity localization deviations, shown in (b), (c), (e) and (f).

Figures 12, 13 and 14 are another three experiment results. For these fingerprint examples, the results show that our approach combing WMCW with the hierarchical smoothing method is capable to extract the information of ridge OF reliably and it is more robust against singularity deviation in comparison with the other two gradient based methods.

In order to objectively evaluate the singularity deviation of the proposed method compared with the state-of-arts approaches, 300 fingerprint images are randomly selected from the database of FVC2004DB1, which the image sizes are fixed to 480 × 480. The genuine singularities are marked by experts as the ground truth for measuring the distance of the detected singularities and the genuine ones. The images contain 358 cores and 135 deltas. We use the method of Ref. [27] for the sake of singularity detection. Euclidean distance is utilized to calculate the distance between the genuine singularities and the detected ones by using Eq. (15):

15 $${\text{d}} = \sqrt {\left( {{\text{x}} - {\text{C}}_{\text{X}} } \right)^{2} + \left( {{\text{y}} - {\text{C}}_{\text{y}} } \right)^{2} }$$

where d indicates the distance, (x, y) is the center pixel of the detected singularity, $({\text{C}}_{\text{X}} ,{\text{C}}_{\text{y}} )$ is the center pixel of the singularity marked by experts.

Table 1 is the distance between the detected cores and the genuine ones. From Table 1 we can observe that the localization deviation incurred by the proposed method is much less than that produced by the enhanced gradient based method (EG) and the gradient-based voting method (GV). Most of the deviation distances of the test fingerprint images by using the proposed method are within 5 pixels. Table 2 is the distance between the detected deltas and the genuine ones. In most cases, deltas locate far away from the center of the image and furthermore the OF close to delta is more stable than that close to the core, so it is not prone to be distorted by smoothing. Table 2 illustrates that the deviation distance of most deltas from the enhanced gradient based method (EG) and the gradient-based voting method (GV) is within 5 pixels. However the proposed method can achieve better result that the deviation distances of most deltas are within 3 pixels. Therefore, we can conclude the proposed method obtains less singularity localization deviation compared to the state-of-arts algorithms.

**Table 1:** The distance between the detected cores and the genuine ones (pixels)

	EG + hierarchical smoothing	GV + hierarchical smoothing	The proposed WMCW + hierarchical smoothing
≥ 15	58	49	31
(10, 15]	132	147	72
(5, 10]	112	104	112
≤ 5	56	76	143

**Table 2:** The distance between the detected deltas and the genuine ones (pixels)

	EG + hierarchical smoothing	GV + hierarchical smoothing	The proposed WMCW + hierarchical smoothing
(5, 10]	17	12	15
(3, 5]	81	72	78
≤ 3	25	41	42

In a word, three experiments results show that the proposed gradient based algorithm is more reliable for the estimation of the ridge information for fingerprint OF and is more accurate in preserving the singularity localization.

## Conclusions

In this paper, a gradient based algorithm, which uses a weighted multi-scale composite window to adapt the scales of the blocks, has been proposed. In order to correct the spurious ridges and preserve the genuine location of singular points, we refine the OF by using a hierarchical smoothing strategy. To verify the performance of the proposed method, three experiments are designed to test the proposed algorithm together with other popular gradient based methods on real fingerprint images,which are selected from different categories and all are suffered from obvious noise effects. The experiment results obtained show that the proposed method is superior with respect to reliable OF construction and avoiding singularity localization deviation.

## Abbreviations

OF: orientation field
CG: conventional gradient based method
EG: enhanced gradient based method
GV: gradient based voting method
WMCW: weighted multi-scale composite window

## References

[1] Sherlock B, Monro D (1993). A model for interpreting fingerprint topology. Pattern Recognit.

[2] Vizcaya P, Gerhardt L (1996). A nonlinear orientation model for global description of fingerprints. Pattern Recognit.

[3] Gu J, Zhou J, Zhang D (2004). A combination model for orientation field of fingerprints. Pattern Recognit.

[4] Zhou J, Gu J (2004). Modeling orientation fields of fingerprint with rational complex functions. Pattern Recognit.

[5] Wang Y, Hu J, Phillips D (2007). A fingerprint orientation model based on 2d fourier expansion (FOMFE) and its application to singular-point detection and fingerprint indexing. IEEE Trans Pattern Anal Mach Intell.

[6] Zhou J, Chen F, Gu J (2009). A novel algorithm for detecting singular points from fingerprint images. IEEE Trans Pattern Anal Mach Intell.

[7] Ram S, Bischof H, Birchbauer J (2010). Modeling fingerprint ridge orientation using legendre polynomials. Pattern Recognit.

[8] Liu M, Liu S, Zhao Q (2014). Fingerprint orientation field reconstruction by weighted discrete cosine transform. Inf Sci.

[9] Karu K, Jain A (1996). Fingerprint classification. Pattern Recognit.

[10] Jain AK, Prabhakar S, Hong I (1999). A multichannel approach to fingerprint classification. IEEE Trans Pattern Anal Mach Intell.

[11] Kass Michael, Witkin Andrew (1987). Analyzing oriented patterns. Computer Vision, Graphics, and Image Processing.

[12] Rao AR, Jain RC (1992). Computerized flow field analysis: oriented texture fields. IEEE Trans Pattern Anal Mach Intell.

[13] Bazen A, Gerez S (2002). Systematic methods for the computation of the directional fields and singular points of fingerprints. IEEE Trans Pattern Anal Mach Intell.

[14] Jiang X (2005). On orientation and anisotropy estimation for online fingerprint authentication. IEEE Trans Signal Process.

[15] Mei Y, Sun HJ, Xia DS (2009). A gradient-based combined method for the computation of fingerprint’s orientation field. Image Vis Comput.

[16] Mei Y, Cao G, Sun HJ (2012). A systematic gradient-based method for the computation of fingerprint’s orientation field. Comput Electron Eng.

[17] Bian W, Luo Y, Xu D (2014). Fingerprint ridge orientation field reconstruction using the best quadratic approximation by orthogonal polynomial in two discrete variables. Pattern Recognit.

[18] Cavusoglu A, Gorgunoglu S (2008). A fast fingerprint image enhancement algorithm using a parabolic mask. Comput Electron Eng.

[19] Wang Y, Hu J, Schroder H. A gradient based weighted averaging method for estimation of fingerprint OF. In: Proceedings of digital imaging computing: techniques and applications. Queensland. 2005.

[20] Srinivasan VS, Murthy NN (1992). Detection of singular points in fingerprint images. Pattern Recognit.

[21] Zhang WC, Zhao YL, Breckon TP (2017). Noise robust image edge detection based upon the automatic anisotropic Gaussian kernels. Pattern Recognit.

[22] Jain A, Hong L, Bolle R (1997). On-line fingerprint verification. IEEE Trans Pattern Anal Mach Intell.

[23] Wang Y, Hu J, Han F (2007). Enhanced gradient-based algorithm for the estimation of fingerprint orientation fields. Appl Math Comput.

[24] Hou Z, Yau WY (2010). A variational formulation for fingerprint orientation modeling. Pattern Recognit.

[25] Liu M, Jiang X, Kot AC (2005). Fingerprint reference-point detection. EURASIP J Adv Signal Process.

[26] Bian WX, Ding SF, Xue Y (2017). Combining weighted linear project analysis with orientation diffusion for fingerprint orientation field reconstruction. Inf Sci.

[27] Wang L, Bhattacharjee N, Srinivasan B. A novel technique for singular point detection based on Poincaré index. In: International conference on advances in mobile computing and multimedia. ACM. 2011. pp. 12–18.

